# A personalized, phenomenological semi-structured interview for diagnostic assessment in young psychiatric patients

**DOI:** 10.1192/j.eurpsy.2026.10333

**Published:** 2026-07-31

**Authors:** I.-M. Mølstrøm

**Affiliations:** Psykiatrien East, Region Zealand, Research Center for Psychopathology, Diagnosis and Early Detection of Psychosis, Roskilde, Denmark

## Abstract

**Abstract:**

Phenomenological psychopathology offers a framework for understanding mental disorders through patients’ subjective experience. While this perspective has gained renewed attention in adult psychiatry, it remains marginal in child and adolescent psychiatry, where diagnostic work is often complicated by developmental variability, evolving reflective capacities, and overlapping symptom presentations.

This presentation orients around the personalized, phenomenologically informed semi-structured interview, and discusses its relevance for early detection in young psychiatric patients.

In everyday clinical practice, early signs of severe mental illness in young patients may present in diffuse and ambiguous ways. Clinicians are faced with uncertainty about whether observed difficulties reflect e.g. transient developmental distress or emerging psychotic disorders. This diagnostic ambiguity contributes to under-recognition and delayed identification of severe mental illness, which in turn is associated with poorer outcomes and increased risk of adverse clinical and social consequences.

Current assessment practices oriented toward symptom checklists and standardized formats, risk overlooking diagnostically decisive alterations in subjective experience present in emerging severe psychopathology. In young patients, clinically central disturbances may not present as readily nameable contents (e.g., specific fears or beliefs), but as alterations in the *form* of experience.

From a clinical perspective, the diagnostic interview is not merely a tool for collecting symptoms, but a central clinical instrument for understanding the patient’s experience, context and suffering. A personalized, phenomenologically informed semi-structured interview allows clinicians to explore not only what the patient reports when asked, but how experiences are lived, articulated, and situated within the patient’s developmental and life context.

The aim is to show and discuss how a phenomenological approach can strengthen clinical judgment and support diagnostic decision-making in child and adolescent psychiatry.

**Disclosure of Interest:**

None Declared

